# Usefulness of urinary NGAL levels during an operation in a patient with septic shock and acute kidney injury for determining postoperative initiation of renal replacement therapy: a case study

**DOI:** 10.1186/s40981-018-0170-5

**Published:** 2018-04-19

**Authors:** Eiko Wada, Kohei Suganuma, Shigehiro Shibata, Kosei Otaka

**Affiliations:** 1 Department of Anesthesia, Omagari Kosei Medical Center, 8-65 Torimachi, Daisen, 014-0027 Japan; 2 Department of Anesthesia and Intensive Care Medicine, Akita University Graduate School of Medicine, Akita, Japan

**Keywords:** Urinary neutrophil gelatinase-associated lipocalin (uNGAL), Acute kidney injury (AKI), Septic shock, Continuous hemodiafiltration (CHDF)

## To the editor

**Fig. 1:**
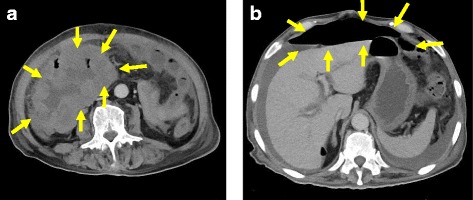
**a** Image of a small intestinal tumor in our patient. The arrows indicate a small intestinal tumor. **b** Image of contrast-enhanced computed tomography (CT) in our patient. The arrows indicate free air

**Fig. 2:**
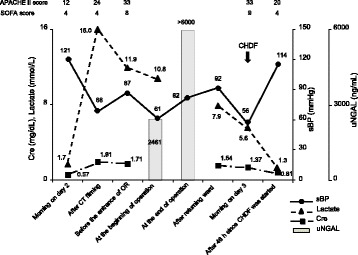
Changes in systolic blood pressure and mediators of AKI and septic shock, such as serum Cre, uNGAL, and serum lactate levels. Cre, creatinine; uNGAL, urinary neutrophil gelatinase-associated lipocalin; sBP, systolic blood pressure; OR, operating room; CHDF, continuous hemodiafiltration; APACHE II score, Acute Physiology and Chronic Health Evaluation II score; SOFA, Sequential Organ Failure Assessment; CT, computed tomography

Although urinary neutrophil gelatinase-associated lipocalin (uNGAL) is useful as a prognostic tool for initiating renal replacement therapy (RRT) [1], determining the timing of initiating RRT for septic shock with acute kidney injury (AKI) may be difficult. Therefore, we attempted to measure uNGAL levels during an operation to determine and prepare the initiation of postoperative RRT. We experienced a case of high uNGAL levels during an operation on a 78-year-old man who underwent emergency loop colostomy due to acute diffuse peritonitis. After open biopsy for a small intestinal tumor (Fig. 1a) on day 1, oliguria and reduced systolic blood pressure were observed on day 2. Gastrointestinal perforation was suspected because contrast-enhanced computed tomography revealed free air (Fig. 1b). Levels of uNGAL at the beginning and at the end of the operation were 2461 and > 6000 ng/mL, respectively (Fig. 2). Reductions in lactate and creatinine (Cre) levels, oliguria, and reduced systolic blood pressure were observed again on day 3. Continuous hemodiafiltration (CHDF) was initiated based on uNGAL levels with failure in RIFLE classification, and the patient’s vital signs improved within the normal range.

Despite successful recovery using CHDF for septic shock with AKI, postoperative CHDF was deferred until the morning of the following day due to reduced Cre and lactate levels. In current clinical practice, AKI is diagnosed by measuring Cre and/or blood urea nitrogen levels, but these markers are insensitive and late indicators of AKI [2]. Previous studies demonstrated that uNGAL levels for AKI were increased within 2 h, and a diagnosis using Cre levels was delayed by 1 to 3 days [3, 4], which may be consistent with our case. Alternatively, perioperative management, such as oxygenation by anesthesia and/or improvement of the circulation during the operation by sufficient fluid administration, potentially caused difficulties in increased lactate or Cre levels after the operation. Further studies are necessary to determine this issue.

uNGAL may be a useful indicator for septic AKI because it is increased by not only AKI but also by inflammation [5, 6]. Although the cutoff value of uNGAL levels for predicting AKI is 193.2 ng/mL (AUC = 0.837) [3], the median value of uNGAL in patients with septic shock without AKI is 471 ng/mL [7]. Therefore, differentiating between AKI and septic shock without AKI by measuring only uNGAL levels is difficult. However, once the septic shock is complicated by AKI, the median value of uNGAL increases to 803 ng/mL [7]. CHDF may also be useful for a septic shock as well as AKI [8]. We used polymethylmethacrylate(PMMA) as a membrane of CHDF, and this membrane continuously and efficiently removes various cytokines from the blood [9]. Therefore, CHDF might, at least in part, have led to the recovery of our patient from AKI and septic shock.

Early evaluation of uNGAL levels during an operation may be useful information for prompt determination and preparation of postoperative initiation of RRT in patients with septic shock accompanied by AKI when lactate or Cre levels might be undetectable after perioperative management.

## Abbreviations

AKI: Acute kidney injury
CHDF: Continuous hemodiafiltration
Cre: Creatinine
PMMA: Polymethylmethacrylate
sBP: Systolic blood pressure
uNGAL: Urinary neutrophil gelatinase-associated lipocalin
